# Bioprocessed Wholegrain Spelt Flour Improves the Quality and Physicochemical Characteristics of Wheat Bread

**DOI:** 10.3390/molecules28083428

**Published:** 2023-04-13

**Authors:** Marjeta Mencin, Nika Markanovič, Maja Mikulič Petkovšek, Robert Veberič, Petra Terpinc

**Affiliations:** 1 Department of Food Science and Technology, Biotechnical Faculty, University of Ljubljana, Jamnikarjeva 101, SI-1111 Ljubljana, Slovenia; marjeta.mencin@bf.uni-lj.si (M.M.);; 2 Department of Agronomy, Biotechnical Faculty, University of Ljubljana, Jamnikarjeva 101, SI-1111 Ljubljana, Slovenia; maja.mikulic-petkovsek@bf.uni-lj.si (M.M.P.); robert.veberic@bf.uni-lj.si (R.V.)

**Keywords:** bioprocessed bread, rheological properties, sensory properties, extractable and bound phenolics, antioxidant activity, HPLC-MS

## Abstract

In the present study, the partial substitution of common white wheat flour for a bread recipe with variously bioprocessed wholegrain spelt was investigated. The addition of 1% and pasteurised 5% “germinated + enzymatic treated” spelt flour to wheat flour significantly improved the specific volume of the bread, but their texture profile analysis and sensory evaluation were not satisfactory. A higher percentage of added bioprocessed spelt flour darkened the colour of the bread. Breads with the addition of more than 5% of bioprocessed spelt flour were unacceptable in terms of quality and sensory parameters. The highest extractable and bound individual phenolics were found in breads with 5% “germinated + fermented” spelt flour (GFB5) and 5% pasteurised “germinated + enzymatic treated” spelt flour (GEB5P). A strong positive correlation was determined between *trans*-ferulic acid and TPC and DPPH^•^ radical scavenging activity. The GEB5P bread showed the highest increase in extractable and bound *trans*-ferulic acid content, by 320% and 137%, respectively, compared to the control bread. Principal component analysis showed differences between the control bread and enriched breads in terms of their quality, sensory and nutritional properties. Breads with 2.5% and 5% “germinated + fermented” spelt flour had the most acceptable rheological, technological and sensory characteristics, in addition to a substantial improvement in their antioxidant content.

## 1. Introduction

Wheat (*Triticum aestivum* L.) is an important crop in most of the world, accounting for between 20% and 50% of the total intake of calories. Furthermore, wheat flour is most commonly used for bread-making because of its gluten protein fraction, which permits the retention of CO_2_ in the viscoelastic dough matrix [1]. An attractive and healthier alternative for bread-making than wheat, with higher nutritional value, is its ancient subspecies, spelt (*Triticum spelta* L.) [2]. In particular, spelt appears to have a higher protein content, higher lipid content, favourable fatty acid profile, and a higher content of various nutrients compared to wheat [3]. In general, spelt bread is characterised by a lower specific volume, darker crumb and crust colour and higher firmness. Spelt gluten is also characterised by higher extensibility and lower elasticity, which generally results in weaker and stickier dough compared to common wheat [3,4]. To solve the quality problems associated with spelt flour, the use of blends with wheat flour has been suggested [5].

Epidemiological studies have linked the consumption of wholegrain products with health benefits in relation to the development of chronic diseases [6]. These effects are not attributed to an individual compound but to the combined action of dietary fibre and bioactive compounds, such as phenolics [7]. Phenolics can be found in cereal seeds in extractable (free or conjugated) and bound forms. In *Triticum* seeds, most phenolics are covalently bound to structural components of the cell wall (cellulose, arabinoxylans, lignin, structural proteins) [8]. According to our previous study, spelt seeds contain the highest amount of ferulic acid, followed by *p*-coumaric, caffeic and *p*-hydroxybenzoic acids [9]. Several strategies have been reported for increasing the content and accessibility of phenolics in bread, one of which is the use of bioprocessing techniques in bread ingredients before bread production [10]. In our previous studies, we reported that the content of extractable phenolics in spelt seeds could be significantly increased with bioprocessing techniques (germination, fermentation, enzymatic treatment), alone or in combination [9,11,12]. In our previous study [11], whose aim was to find the stress factors that could contribute to the increase in phenolic content during germination, we investigated the influence of different amounts of water, salinity, osmolarity, germination temperature, and mechanical damage. In total, we tested more than 30 different abiotic stress conditions-both individually and in combination. We concluded that germination under combined stress of 25 mM NaCl and 50 mM sorbitol without subsequent mechanical stress had the greatest overall effect on total phenolic content and scavenging activities against different free radicals [11]. The reason why the fermentation in the present study was performed with *Saccharomyces cerevisiae* was based on the optimisation experiments previously performed on raw, germinated, and enzymatically-treated spelt seeds [9]. We tested the effect of lactic acid (*Lactobacillus plantarum*), alcoholic (*S. cerevisiae*) and spontaneous fermentation individually and in combination and concluded that the highest extractable and bound individual phenolic contents and antioxidant activities of the extracts were obtained in germinated spelt seeds fermented with *S. cerevisiae* [9]. Previously, we also investigated the effect of cellulase, xylanase, esterase, α-amylase, and protease (alone or in combination) on the enhanced release of phenolics from spelt seeds [12]. We made decisions about the specific enzymes, their concentrations, and the conditions under which they were used based on the optimisation experiments previously performed. In addition, the co-effect of optimal enzymatic treatment, germination or fermentation on the content of spelt phenolics and their antioxidant activity was investigated. Since we were able to demonstrate great differences in the content and accessibility of phenolics between only germinated and enzymatically-treated germinated seeds, we strongly believe that the addition of exogenous enzymes in germinated seeds (despite the relatively high amount of endogenous enzymes) is quite justified [12]. Other studies also have reported that enzymatic treatment and fermentation have the potential to improve nutritional and technological properties, mainly improving the volume, crumb texture and shelf life of the final product [13,14,15,16]. Fermentation activates endogenous enzymes of seeds, which, together with microbial enzymes, modifies the state of the fibre in seeds, resulting in the solubilisation of arabinoxylans and a slight degradation of the insoluble fibre [17]. Similarly, Angelino et al. [10] reported that the application of external enzymes or exploitation of a synthesis of endogenous enzymes through germination is a promising approach for converting bread-bound phenolics into extractable form. In addition to improving the textural properties of bioprocessed breads, sourdough fermentation of bran has also been shown to increase the concentration of free phenolic acids in the final product [14,18]. Furthermore, Katina et al. [19] showed that enzymatic treatment and fermentation of wheat bran effectively improve the texture, sensory quality and stability of breads enriched with 20% wheat bran. Enzymes that hydrolyse starch and non-starch carbohydrates are widely used in the bread-making industry as bread improvers. It has been reported that enzyme hydrolysis of non-starch polysaccharides leads to an improvement in the rheological properties of dough, the bread-specific volume and crumb firmness [20].

The aims of this study were: (i) to investigate the potential of using bioprocessed spelt flour in a wheat bread recipe to improve the quality and sensory characteristics; and (ii) to evaluate the effects of added bioprocessed spelt flour on the total phenolic content (TPC), phenolic profile and antioxidant activity of enriched wheat bread.

## 2. Results and Discussion

### 2.1. Rheological Properties of Dough

**Figure 1:**
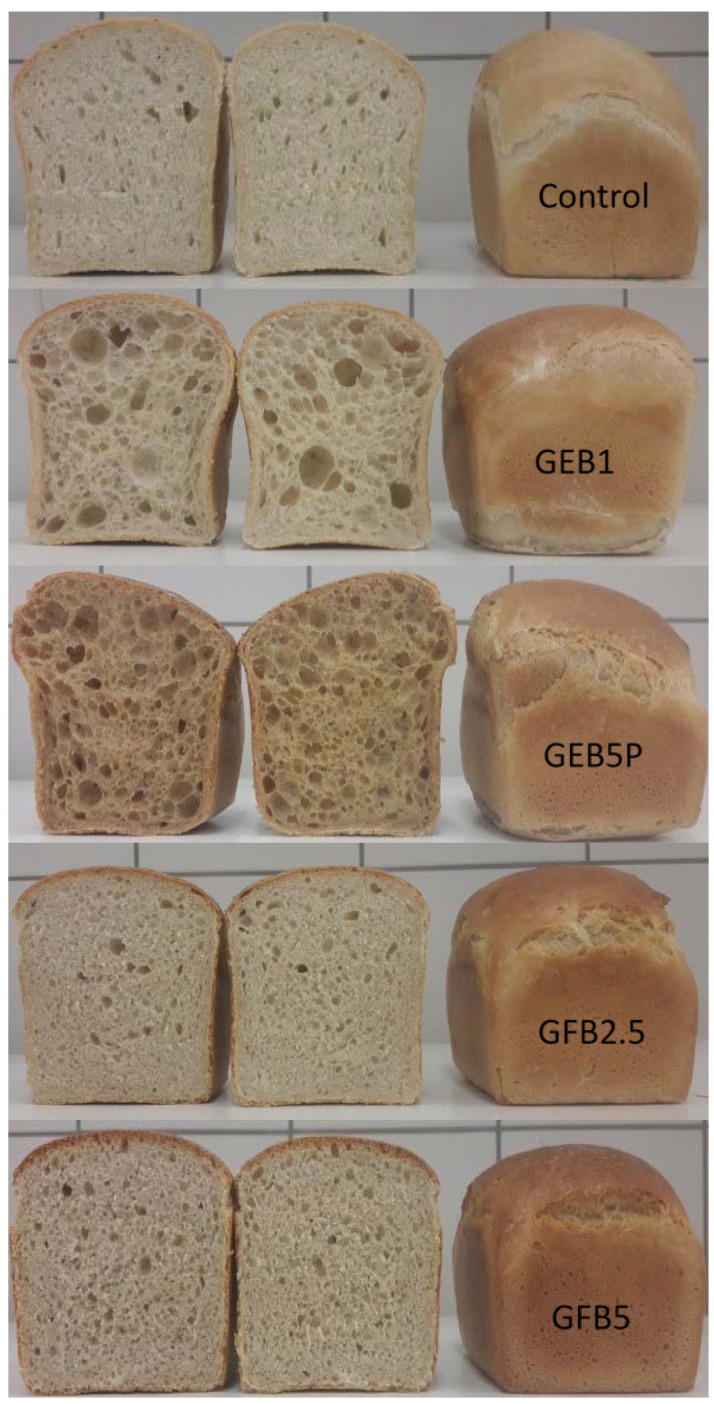
Photographs of bread prepared from white wheat flour (control), with 1% of »germinated + enzymatic treated« spelt flour (GEB1), with 5% of pasteurised »germinated + enzymatic treated« spelt flour (GEB5P), with 2.5% of “germinated + fermented” spelt flour (GFB2.5), or with 5% of “germinated + fermented” spelt flour (GFB5).

The farinographic parameters are presented in Table 1 and Figure S1. The white wheat flour required 60.1% water absorption to reach the optimum dough consistency (500 FU), while water absorption was lower for the wheat flour substituted with 2.5% and 5% of “germinated + fermented” wholegrain spelt flour (56.8% and 56.0%, respectively). Slightly lower water absorption capacity of spelt flour than for common wheat was also found in a comparative study between wheat and spelt [4], which revealed significant differences in the composition of gluten elemental components, i.e., gliadins and glutenin subunits, as well as in the gliadin/glutenin ratio. While in spelt gluten, gliadins predominate as a very sticky monomeric plasticiser, common wheat gluten is mainly determined by glutenins as a networking polymeric factor. Moreover, Bae et al. [21] reported that the addition of xylanase and amylase to wholegrain wheat flour reduces its water absorption. The enzymatic degradation of damaged arabinoxylans and starch during mixing probably affected the dough’s hydration and mixing properties [21]. Our bioprocessed spelt flour had a high concentration and activity of hydrolytic enzymes due to the activation and synthesis of enzymes during germination and the involvement of enzymes from microorganisms during fermentation. In addition, lower water absorption could lead to a hard dough since the gluten network does not completely form without sufficient water absorption, resulting in an inability to hold air during fermentation, which also causes a lower bread volume (Figure 1). The addition of bioprocessed wholegrain spelt flour resulted in a reduction in dough development and stability times. These results may indicate that the presence of fibre-rich bran in the bioprocessed wholegrain spelt flour leads to the formation of a weaker gluten network and is less stable during extended mixing. This is probably due to a series of physical and chemical interactions that directly affect the stability of the dough since this depends on the number of bonds between the protein molecules present in the gluten and the strength of these bonds [22]. Studies [23,24] have reported that wholegrain flours generally show higher water absorption and development time than white wheat flour. The rheological behaviour of enriched wheat flours in our study was apparently the result of both gluten and fibre qualities and quantities. Dough stability was significantly lower in the GFB2.5 sample. Interestingly, the stability time of the GFB5 dough was similar to that of the control (wheat) dough. Dough made from 2.5% and 5% “germinated + fermented” wholegrain spelt flour showed a significantly higher degree of softening than dough made from wheat flour. Higher values of the degree of softening indicate that the dough is not able to sustain long mechanical processing and, consequently, indicate a poorer quality of the flour. It is of note that the farinograph quality number showed that the GFB5 dough was of better quality than the dough to which 2.5% “germinated + fermented” spelt flour had been added.

The extensiographic properties of the doughs are presented in Table 1 (only after 135 min resting time) and Figure S2 (through 45, 90 and 135 min resting time periods). The extensiographic energy of doughs made with 2.5% and 5% “germinated + fermented” spelt flour was lower than that of the control dough. Lower energy means a lower bread volume (Figure 1) and results in a dough with lower gas-retaining capacity, which may be caused by an impairment of gluten properties due to increased protease activities [25]. Extensibility, a measure of the dough’s tendency to extend during proofing, was significantly increased when bioprocessed wholegrain spelt flour was used. In contrast, the resistance to extension decreased when bioprocessed wholegrain spelt flour was used. A similar trend was observed for the maximum resistance to extension. This is probably due to a weakening of the dough and can be explained by the physical interference mechanism that the presence of the outer layers of the wheat grain provides to the gluten. Furthermore, Altinel & Ünal [26] reported that endoxylanase reduces the resistance to extension of whole wheat dough because it softens the dough.

### 2.2. Characteristics of the Breads Produced

#### 2.2.1. Specific Volume, Colour, Texture

The quality characteristics of bread samples were evaluated 3 h after baking and are presented in Table 2. The results show that the addition of bioprocessed flour had an effect on the specific volume of the bread. The specific volume was significantly improved with the addition of 1% “germinated + enzymatic treated” spelt flour (GEB1) and with the addition of 5% pasteurised “germinated + enzymatic treated” spelt flour (GEB5P) by 16% and 18%, respectively, compared to the control. The addition of 30% “germinated + fermented” spelt flour (GFB30) showed the highest decrease in specific volume, by 47% compared to the control. Furthermore, the quality of bread enriched with 30% “germinated + enzymatic treated” spelt flour (GEB30) was unacceptable, and we could not measure its specific volume. Breads enriched with 5% of bioprocessed spelt flour (GFB5, GEB5) showed a decrease in specific volume of about 22%, probably due to the mechanical disruption of the gluten network by the bioprocessed flour. Our results clearly indicate that substituting refined flour with bioprocessed beyond 1% leads to a significant decrease in the specific volume of the breads. The only exception was when pasteurisation was used, which made it possible to inactivate the enzymes present in spelt when bioprocessed flour was added to the bread formulation. Bioprocessing techniques increase biosynthesis and the activity of enzymes in spelt flour and thus have a positive effect on the specific volume of breads, but only up to a certain percentage of added bioprocessed spelt flour. Bioprocessed flour modified the balance between soluble and insoluble fibres in the analysed breads. During this process, the hydrolysis of water-nonextractable arabinoxylan from wheat-spelt dough occurred, and the amount of water-extractable arabinoxylan increased [27]. Extractable arabinoxylans have a positive effect on the specific volume of bread since this fibre source can stabilise the gas cells by increasing the viscosity of the water phase in the dough. Moreover, the α-amylases present in the bioprocessed flour degrade the damaged starch into small dextrins, allowing the yeast to work continuously during dough fermentation, resulting in improved bread volume. Amylase functionality under increased specific volume may be related to reduced dough viscosity during starch gelatinisation, prolonging the rising process in the oven [28]. Recent studies have reported that the use of bioprocessed flour improves the bread-specific volume and crumb texture [14,17,29]. A decrease in bread volume when using a greater extent of bioprocessed flour, on the other hand, can be explained by an impairment of gluten properties due to increased protease activities, resulting in a dough with lower gas-retaining capacity [25] and excessive liquefaction and dextrinisation, yielding bread with a wet, sticky crumb [30].

In terms of bread crust colour, the L* value (lightness) of the control was significantly higher than that of the bioprocessed breads. With a higher percentage of bioprocessed spelt flour in the bread recipe, the lightness of the bread’s crust decreased (Figure 1, Table 2). On the other hand, the crust of control bread showed a significantly lower a* value than those of bioprocessed breads. The highest a* values were shown by GFB5, GEB10P and GEB5P breads. A higher addition of bioprocessed spelt flour decreased the yellowness, indicated by the b* value, as shown in Table 2. The crust colour of breads has been greatly affected by replacing wheat flour with bioprocessed spelt flour. The ΔE value is used to measure the overall colour difference perceivable by the human eye. The ΔE values can be interpreted as follows: ΔE < 1, colour differences were not visible to the human eye; 1 < ΔE < 3, colour was not noticeably different to the human eye; ΔE > 3, colour differences were noticeable to the human eye [31]. The ΔE values showed that a higher percentage of added bioprocessed spelt flour resulted in a higher total colour change of breads. These colour changes can be attributed to the increased α-amylase activity in bioprocessed flour, which hydrolyses starch and consequently increases the concentration of reducing sugars such as glucose, fructose etc., which can participate in the Maillard reaction or be caramelised during baking, giving the bread crust a brown colour [3]. Moreover, the reducing sugars and amino acids released during germination have a significant and positive effect on the profile of Maillard reaction products released during baking. The darker bread colour with added bioprocessed spelt flour can also be attributed to the naturally darker colour of bioprocessed whole spelt flour compared to white wheat flour.

The change in crumb firmness was measured only in three bread samples (control, GFB5, GFB2.5); measurements of other samples could not be obtained because the crumbs were too sticky. The supplement of 5% wheat flour with “germinated + fermented” spelt resulted in a 111% increase in firmness, while flour replacement of 2.5% had no statistically significant impact on it. The wet sticky crumbs of other bread samples can be explained by the excessive hydrolysis of starch, proteins and arabinoxylan, resulting in the release of an excess of water previously bound to these polymers. The increase in enzyme activity was not only a consequence of the presence of microorganisms or the addition of exogenous enzymes but was also caused by the activation of endogenous enzymes during the germination of spelt seeds. Excessive darkening of the bread crust and stickiness of the dough were associated with increased activity of α-amylases and xylanases. It is known that the profound degradation of arabinoxylan by exogenous xylanases and the release of water previously bound to arabinoxylan can lead to very poor dough manageability [25]. Furthermore, the elevated protease activity in bioprocessed flour can result in the additional release of water due to the degradation of proteins during dough processing. Other studies have also reported a negative impact of a higher addition (more than 10%) of bioprocessed flour on the quality parameters of bread [32,33,34]. Bioprocessed flours in proper quantities can be used as an alternative to conventional flour improvers such as enzymes and malt.

#### 2.2.2. Sensory Evaluation

Sensory analysis was carried out three hours after baking (Table 3). Overall, the control bread, which was made without bioprocessed spelt flour, was characterised by a score of 0 and served as a basis for comparison. The sensory characteristics were evaluated with scores ranging from −3 to +3. In relation to the colour of the crust and crumb, a score of −3 meant the lightest colour, and +3 meant the darkest colour. For crumb porosity, a score of −3 indicated the smallest pores and tight, dense crumbs, 0 indicated uniform porosity, while a score of +3 indicated large pores and uneven porosity. In relation to the bread texture and aroma (odour, taste) parameters, a score of −3 meant a poor, firm, less soluble texture and less intense and characteristic aroma, while a score of +3 meant a soft, soluble texture and the most intense and characteristic aroma. The breads GFB30, GEB30, GEB10P and GEB5 were unacceptable in terms of quality parameters, so these breads were excluded from sensory evaluation. They had no crumb; the inside of the bread was just a big hole (Figure S3). Extensive starch degradation by excess enzyme activity during baking is likely to weaken the starch-protein matrix, resulting in a collapse of the crumb structure. In addition, a large decrease in the viscosity of the dough’s aqueous phase can lead to lower foam stability of the dough and hence its collapse [25]. The breads with added “germinated + enzymatic treated” spelt flour (GEB1, GEB5P) possessed larger bubbles with thicker cells and more holes in the crumb, and they had uneven porosity (Figure 1). These two breads had a very sticky and wet crumb, and they were lumpy in the mouth. According to Sahi & Little [35], thicker cell walls affect the visual and edible quality, imparting a grey-looking crumb and a firm mouthfeel. The crumb was not elastic, and for the reasons mentioned above, the sensory quality of the GEB1 and GEB5P breads was unacceptable. The GEB5P bread had a sweet taste untypical for bread, and the GEB1 bread was not suitable for tasting. Similarly, de Almeida et al. [36] reported that enzymatic bioprocessed whole wheat bread samples had greater volume, but their sensory characterisation and acceptance were compromised.

The addition of 5% and 2.5% “germinated + fermented” spelt flour was characterised by a slightly more distinct smell and taste of spelt, as well as a crust taste of cheese and caramel. GFB2.5 and GFB5 had quite good porosity, uniform pores in the crumb, a fairly elastic-flexible crumb and a slightly sticky crumb, but this gives a feeling of freshness. The colour of the crumb was a little darker but still typical of bread. These two breads were pleasant in the mouth, not too sticky, and were preferred among all the bioprocessed breads. In general, the assessors preferred breads enriched with up to 5% “germinated + fermented” spelt flour.

Bioprocessing did not improve the texture score of the breads. All sensory scores decreased when enzymatic treatment was combined with germination of spelt seeds, leading to the rejection of these breads in terms of appearance, flavour, texture, overall liking and, consequently, purchase intention. Although enzymes have become a promising strategy in baking positively to modify the rheological properties of dough without affecting sensory acceptance, in the present study, the addition of 5% of bioprocessed spelt flour already impacted the characterisation and acceptance of the bread samples, meanwhile breads with 30% substitution of white flour were completely inadequate for sensory assessment. On the other hand, the addition of 2.5% and 5% of “germinated + fermented” spelt flour resulted in bread with acceptable technological characteristics and sensory profile. All sensory properties decreased when the “germinated + enzymatic treated” spelt flour was added to the bread recipe. This is in agreement with the study of de Almeida et al. [36], who reported that enzymatic bioprocessing combined with the addition of green coffee in whole wheat led to the rejection of this bread in terms of appearance, flavour, texture, overall liking and purchase intention.

Furthermore, de Almeida et al. [36] reported that consumers are more likely to accept foods that contain information about the presence of health-promoting substances and are less receptive to foods with an unhealthy appeal, such as industrialised products. These data are also in agreement with the proposal of Sajdakowska et al. [37], who suggested that information about the health benefits of foods with nutritional and functional improvements increases consumer knowledge and positively influences their acceptance.

### 2.3. Total Phenolic Content and Antioxidant Activity in Breads

**Figure 2:**
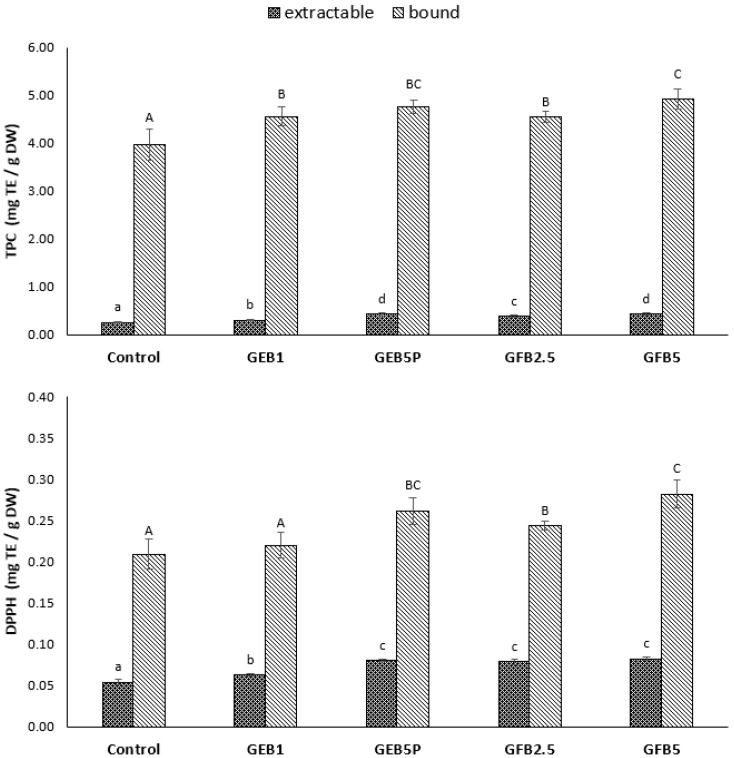
Total phenolic content (TPC) and DPPH^•^ scavenging activity (DPPH) for the extractable and bound fractions of control and bioprocessed breads. Data are means ± SD. Different small letters indicate statistically significant differences between the extractable phenolics (*p* < 0.05; Duncan’s multiple range tests). Different capital letters indicated statistically significant differences between the bound phenolics (*p* < 0.05; Duncan’s multiple range tests). Control: white wheat bread; GEB1: wheat bread supplemented with 1% “germinated + enzymatic treated” spelt flour; GEB5P: wheat bread supplemented with 5% pasteurised (30 min, 85 °C) “germinated + enzymatic treated” spelt flour; GFB2.5: wheat bread supplemented with 2.5% “germinated + fermented” spelt flour; GFB5: wheat bread supplemented with 5% “germinated + fermented” spelt flour; mg TE/g DW: mg Trolox equivalents per g dry weight.

In our study, the TPC and antioxidant activity were determined in the five breads with the most promising characteristics. The results for TPC and antioxidant activity assays of extractable and bound phenolics are shown in Figure 2. The wheat breads enriched with bioprocessed spelt flour had higher extractable and bound TPCs than the control bread. Extractable TPC increased the most in breads with 5% “germinated + fermented” (GFB5) and 5% pasteurised “germinated + enzymatic treated” (GEB5P) spelt flour, by 67% and 64%, respectively, compared to the control, with GEB5P having an inappropriate crumb firmness. The increase in the extractable TPC was closely related to the percentage of added bioprocessed spelt flour in the bread recipe. The addition of “germinated + fermented” spelt flour promoted a similar effect to that of depolymerising arabinoxylans, which can be explained by the fact that yeast and other microorganisms produce compounds with depolymerising properties. Moreover, germination triggers the enzymatic activity of sprouting seeds, leading to the degradation of starch and non-starch polysaccharides and proteins, resulting in an increase in reducing sugars, soluble dietary fibre, peptides and amino acids, as well as to the release of the insoluble phenolic compounds covalently bound to cell wall polysaccharides, all affecting higher extractable TPC [38].

The highest increase in bound TPC was also found in GFB5 (24%) and GEB5P (20%) breads compared to the control bread. The increase in bound TPC using “germinated + enzymatic treated” spelt flour was due to the fragmentation of arabinoxylans, which became more soluble and easily accessible during extraction. With respect to the increase of bound TPC, it is relevant to mention that both enzymatic and yeast treatment of germinated spelt flour affects the distribution of phenolics, which may have undergone polymerisation or conjugation reactions. The total (extractable + bound) TPC was thus significantly increased, from 15% in GEB1 to 27% in GFB5 bread compared to the control bread. A considerably higher proportion of bound TPC was observed for all bioprocessed and control bread (more than 90%). Interestingly, despite a 5-fold increase in the added “germinated + enzymatic treated” spelt flour (from 1% to 5%), no increase in bound TPC was achieved. In contrast, with a 2-fold increase to the added “germinated + fermented” spelt flour (from 2.5% to 5%), an increase was achieved, although it was minimal. It seems that the increase in extractable and bound TPCs did not depend on the type of bioprocessed spelt flour but rather on the percentage of bioprocessed flour added and, in the case of “germinated + enzymatic treated” spelt flour, on the pasteurisation.

The increased TPC in bioprocessed breads was expected since bioprocessed flour contains significantly higher extractable and bound TPC than white wheat flour. Specifically, the “germinated + fermented” and “germinated + enzymatic treated” spelt flours contain 157% and 109% higher total (extractable + bound) TPC, respectively, compared to raw spelt flour [9,12]. Cornejo et al. [39] reported that germinated brown rice flour bread showed a 1.5-fold higher total TPC than brown rice flour bread. Anson et al. [40] showed that wheat bran bioprocessed with yeast fermentation in combination with cell wall hydrolytic enzymes increased the free phenolics in wheat breads. Banu et al. [27] showed that enzymatic bioprocessing and sourdough fermentation increased the total TPC of wheat bread. A study by de Almeida et al. [6] reported that enzymatic bioprocessing increased the extractable TPC in whole wheat breads by 1.8-fold. Ktenioudaki et al. [15] showed that free TPC was higher for sourdough breads. They also reported that bound TPC was significantly higher than free TPC, but there were no significant differences among the bread samples.

The obtained results indicate that the antioxidant activity of control bread determined by the DPPH^•^ scavenging activity assay was at 0.054 mg TE/g DW for extractable TPC and at 0.209 mg TE/g DW for bound TPC (Figure 2). The higher antioxidant activity corresponded to the bound phenolics. The addition of bioprocessed flour to wheat bread resulted in a statistically significant increase in the antioxidant activity of extractable TPC. The highest increase in the antioxidant activity of extractable TPC was observed in GEB5P (49%), GFB2.5 (45%) and GFB5 (53%) breads compared to the control bread. Interestingly, the addition of 1% “germinated + enzymatic treated” spelt flour showed no statistical difference in the antioxidant activity of bound TPC compared to the control bread, although there was a statistical difference in bound TPC between GEB1 and the control breads. The highest increase in antioxidant activity of bound TPC was observed in GFB5 bread (35%) compared to the control. The percentage of added bioprocessed flour affects the antioxidant activity of bread, although the differences in the percentages of added bioprocessed flour are better reflected in the TPC. The “germinated + fermented” and “germinated + enzymatic treated” spelt flours showed significantly higher total antioxidant activity, of 272% and 141%, respectively, compared to raw spelt flour [9,12]. The antioxidant activity of bioprocessed breads was therefore expected to be significantly higher than the antioxidant activity of the control bread.

Coefficients of correlation (r) were calculated to explain the relationship between TPCs and the antioxidant activity of extractable and bound fractions. The extractable and bound fractions showed a positive and strong correlation between TPC and antioxidant activity (r = 0.991 and r = 0.880, respectively). These results indicate that phenolics strongly contributed to the antioxidant activity of breads.

The increase in antioxidant activity observed in our research in bioprocessed breads is consistent with research reports that indicate that the antioxidant activity (measured by various assays) increases with the addition of bioprocessed flour. Ktenioudaki et al. [15] reported that sourdough fermentation and the use of enzymes increased antioxidant activity in breads. Amaya Villalva et al. [41] reported that the antioxidant activity in bioprocessed bran breads showed a consistent correlation with TPC, the antioxidant activity being higher in bioprocessed bran breads than in native bran bread. They also found that the majority of antioxidant activity was found in the bound fraction. It is important to point out that the bread-making process can also contribute to the improvement of antioxidant activity, particularly because several reactions can occur during these processes that enable the production of antioxidants [40]. Based on our results, we assume that bioprocessed breads could be developed as functional foods with more effective antioxidant properties.

### 2.4. Phenolic Profile of Breads

**Figure 3:**
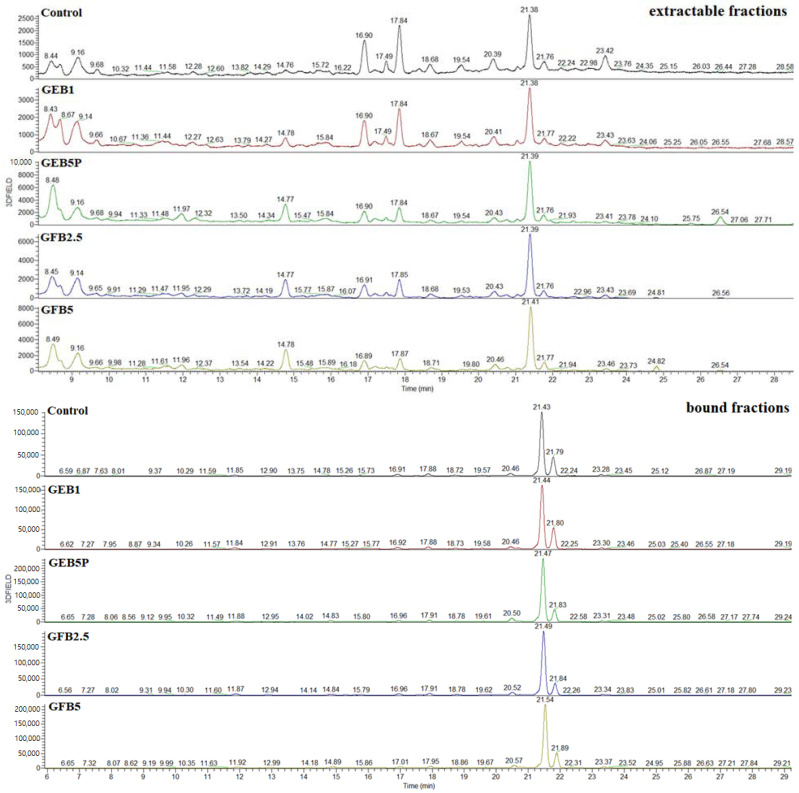
HPLC-MS chromatograms of the extractable and bound fractions of the control and bioprocessed breads (detected at 310 nm). Control: white wheat bread; GEB1: wheat bread supplemented with 1% “germinated + enzymatic treated” spelt flour; GEB5P: wheat bread supplemented with 5% pasteurised (30 min, 85 °C) “germinated + enzymatic treated” spelt flour; GFB2.5: wheat bread supplemented with 2.5% “germinated + fermented” spelt flour; GFB5: wheat bread supplemented with 5% “germinated + fermented” spelt flour.

The effect of adding bioprocessed spelt flour to breads on individual extractable and bound phenolics was evaluated with HPLC-MS (Table 4, Figure 3). Five phenolic acids and four flavonoids were identified in the control and bioprocessed breads. In general, the percentage of added bioprocessed flour had a significant impact on the phenolic acid content. Our results indicated that the addition of bioprocessed spelt flour to white flour contributes to an increase in the extractable phenolic acid content in bread. Ferulic acid was the predominant phenolic identified in breads and accounted for between 62% of total phenolics in the control bread and 73% of total phenolics in GFB2.5 bread. More than 98% of the total (extractable + bound) ferulic acid was bound to cell wall components. The bread enriched with 5% pasteurised “germinated + enzymatic treated” spelt flour (GEB5P) showed the highest increase in the content of extractable and bound *trans*-ferulic acid, by 320% and 137%, respectively, compared to the control bread. Interestingly, the total (extractable + bound) ferulic acid content in “germinated + fermented” spelt flour was higher than in “germinated + enzymatic treated” spelt flour by approx. 9% [9,12], but when we added the same percentage (5%) of these two bioprocessed flours to the bread recipe, the content of total ferulic acid was approx. 9% higher in bread enriched with 5% pasteurised “germinated + enzymatic treated” spelt flour. The total content of *trans*-ferulic acid (extractable + bound) in breads was up to 5-fold, 34-fold, 142-fold and 15-fold higher than that of total *cis*-ferulic, *p-*coumaric, caffeic and *p-*hydroxybenzoic acids, respectively.

The second most abundant phenolic in our analysed breads was *cis*-Ferulic acid. Similar to *trans*-ferulic acid, the highest increase in extractable (250%) and bound (45%) *cis*-ferulic acid content was observed in GEB5P bread compared to the control bread. The type and percentage of added bioprocessed spelt flour had a greater influence on the extractable *cis*-ferulic acid content than on the bound content.

The highest increase in extractable *p-*coumaric acid content was observed in bread enriched with 5% “germinated + fermented” spelt flour, by 42% compared to the control bread. The difference between the content of extractable *p-*coumaric acid in breads enriched with 5% “germinated + fermented” spelt flour and 5% pasteurised “germinated + enzymatic treated” spelt flour was 13%, but the “germinated + fermented” spelt flour had a 24% higher total content of *p-*coumaric acid than “germinated + enzymatic treated” spelt flour [9,12]. The latter can be explained by the complex transformations that take place during dough mixing, proofing and baking, as well as by the application of pasteurisation, which resulted in the inactivity of spelt enzymes (endo- and exogenous) in the dough. Interestingly, the increase in bound *p-*coumaric acid content was much higher, from 66% in GEB1 bread to 449% in GEB5P bread, compared to the control.

For extractable caffeic and *p-*hydroxybenzoic acids, the highest increase was observed in GFB5 bread, by 100% and 159%, respectively, compared to the control bread. This may be because of the content of total caffeic acid in the “germinated + fermented” spelt flour, which was 142% higher than in the “germinated + enzymatic treated” flour. Furthermore, the content of total *p-*hydroxybenzoic acid in “germinated + fermented” spelt flour was 27% higher than in “germinated + enzymatic treated” flour [9,12]. The highest increase in bound *p-*hydroxybenzoic acid was observed in GEB5P bread compared to the control, while there was no statistical difference between GEB5P and GFB5 breads regarding the content of bound caffeic acid.

When compared to the control, the highest relative increase in extractable apigenin hexoside pentoside I (89%), II (125%) and III (37%) was observed in GEB5P bread, while the preparation of GFB5 was equally relevant for the contents of bound apigenin hexoside pentosides I (165%), II (482%) and III (72%). On the other hand, the addition of “germinated + fermented” spelt flour to the bread recipe had a negative impact on the content of extractable unknown C-glycosyl derivative compared to the control bread, while the addition of 5% “germinated + fermented” spelt flour showed the highest increase (713%) in bound C-glycosyl derivative compared to the control.

Interestingly, Amaya Villalva et al. [41] reported that a decrease in the bound fraction of all hydroxycinnamic acids in bioprocessed bran breads compared to native bran bread was consistent with an increase in the free fraction, with the exception of *p-*coumaric acid. They also reported that the free ferulic acid content increased by 69–93% in all bioprocessed bran breads compared to native bran bread. Furthermore, Anson et al. [40] found that fermentation of bran increases the free ferulic acid content of the bread by 3-fold, while a combination of fermentation and enzymatic treatment of bran increases the free ferulic acid content by 8-fold. Ktenioudaki et al. [15] found that the highest concentrations of the individual phenolics identified in the free extracts of all breads enriched with bioprocessed (sourdough fermentation and xylanase treatment) brewer’s spent grain flour were represented by ferulic, protocatechuic, *p-*coumaric and gallic acids. In the bound extracts of the bioprocessed breads, they found only ferulic and *p-*coumaric acids, and the concentrations averaged 387 and 95.5 μg/g DW bread, respectively. Koistinen et al. [42] showed that ferulic acid is the main phenolic acid in wheat bread enriched with native or bioprocessed (enzymatic treatment + yeast fermentation) rye bran. They found that the content of free ferulic acid in bioprocessed bran bread was 10-times higher than in native bran bread, while the content of bound ferulic acid in bioprocessed bran bread was 4% lower than in native bran bread, and the same trend was observed for the content of *p-*coumaric acid.

It is important that with the addition of bioprocessed flour, we increase the content of phenolics in the bread, especially in extractable form, because the bioaccessibility of phenolic acids is mainly associated with the amount of extractable phenolic acids present in bread, and if we want bread to have a positive effect on our health, the phenolics must be bioaccessible. The increase in extractable phenolic acids using yeast fermentation or enzymatic treatment was due to the action of hydrolytic enzymes.

We suggest that *trans*-ferulic, *cis*-ferulic and *p-*coumaric acids are the main contributors to the TPC and antioxidant activity of the extractable fraction. The main contributors for predicting bound TPC and antioxidant activity are *trans*-ferulic, *p-*coumaric and caffeic acid. Among phenolics analysed, *trans*-ferulic acid had the greatest influence on the extractable and bound TPC (r = 0.967 and r = 0.919, respectively) and DPPH^•^ radical scavenging activity (r = 0.929 and r = 0.909, respectively). Bound *cis*-ferulic acid showed a lower correlation with bound TPC and DPPH^•^ radical scavenging activity (r = 0.660 and r = 0.564, respectively).

### 2.5. Principal Component Analysis

**Figure 4:**
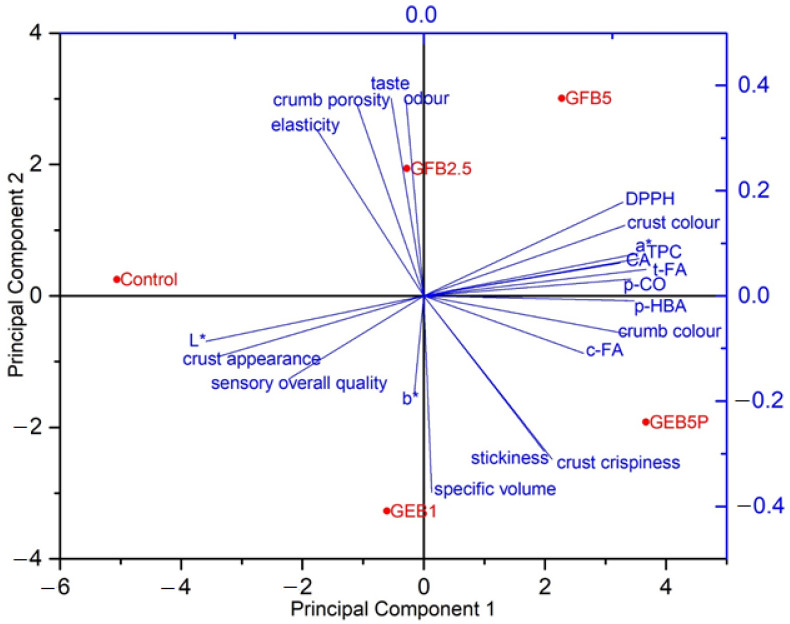
Plot of the variables and bread samples on the first and second principal component axes. Control: white wheat bread; GEB1: wheat bread supplemented with 1% “germinated + enzymatic treated” spelt flour; GEB5P: wheat bread supplemented with 5% pasteurised (30 min, 85 °C) “germinated + enzymatic treated” spelt flour; GFB2.5: wheat bread supplemented with 2.5% “germinated + fermented” spelt flour; GFB5: wheat bread supplemented with 5% “germinated + fermented” spelt flour; L*: lightness; a*: redness; b*: yellowness; t-FA: *trans*-ferulic acid; c-FA: cis-ferulic acid; p-CO: p-coumaric acid; CA: caffeic acid; p-HBA: p-hydroxybenzoic acid; TPC: total phenolic content; DPPH: DPPH^•^ radical scavenging activity.

We performed PCA for further analysis of the obtained data, based on a correlation matrix, using the bread quality and sensory parameters, as well as the results of the total (extractable + bound) TPC and individual phenolic acids and antioxidant activity. In the PCA, as shown in the two-dimensional plot in Figure 4, the first two principal components accounted for 85.8% of the total variance. Bread samples were separated along the first principal component (PC1), which explained 53.3% of the variance in the data, by differences in total TPC, DPPH^•^, *trans*-ferulic acid, *cis*-ferulic acid, *p-*coumaric acid, caffeic acid, *p-*hydroxybenzoic acid, L* and a* values, crust appearance, sensory overall quality, crust and crumb colour. Then PC2, which explained 32.5% of the variance, separated the bread samples based on specific volume, b* value, crust crispiness, crumb porosity, elasticity, stickiness, odour and taste. Projecting the variables onto the plot showed that the control bread was defined particularly by its lightness (L*), crust appearance, sensory overall quality, elasticity and crumb porosity. On the other hand, the control bread had the lowest content of phenolic antioxidants. According to the PCA plot, there was a significant difference in total TPC and individual phenolic acids content between the control bread and bioprocessed breads, with the percentage of added bioprocessed flour having the greatest influence on the content of phenolics and their antioxidant activity against the DPPH^•^ radical.

Plotting the data in a two-dimensional coordinate system (Figure 4) showed that PC2 separated breads enriched with “germinated + enzymatic treated” spelt flour from the control bread and breads enriched with “germinated + fermented” spelt flour. The breads enriched with “germinated + fermented” spelt flour had better crumb elasticity and porosity, as well as better odour and taste than breads enriched with “germinated + enzymatic treated” spelt flour, which had an extremely sticky crumb but the higher specific volume and better crispiness of the crust. GEB1 and GEB5P had the greatest specific volume among the analysed breads, probably due to the use of xylanase in the enzymatic treatment of the germinated spelt flour.

The PC1 separated breads enriched with 5% bioprocessed spelt flour from the control bread and breads enriched with a lower percentage (1% and 2.5%) of bioprocessed spelt flour. Breads with 5% bioprocessed spelt flour had a higher total (extractable + bound) TPC and individual phenolic acids content and higher antioxidant activity against DPPH^•^ radicals and had a darker crust and crumb colour and higher redness.

## 3. Materials and Methods

### 3.1. Materials

Dehulled spelt (*Triticum spelta* L. cv. Ostro) seeds were purchased from a Slovenian producer and were stored in the dark at 1 °C. Absolute methanol, formic acid, sodium hydroxide and sodium carbonate were purchased from Merck (Darmstadt, Germany). DPPH^•^ (1,1-diphenyl-2-picrylhydrazyl radical) reagent, Folin–Ciocalteu reagent, Trolox, *p*-hydroxybenzoic acid, *trans*-ferulic acid, *p*-coumaric acid, caffeic acid, α-amylase (EC 232-560-9) from *Bacillus amyloliquefaciens* (enzyme activity 250 U/g) and protease (EC 232-752-2) from *Aspergillus oryzae* (enzyme activity 500 U/g) were from Sigma-Aldrich (Steinheim, Germany). Cellulase (EC 3.2.1.4) from *Talaromyces emersonii* (enzyme activity 70 U/mg), xylanase (EC 3.2.1.8) from *Aspergillus niger* (enzyme activity 79 U/mg) and feruloyl esterase (EC 3.1.1.73) from rumen microorganism (enzyme activity 30 U/mg) were from Megazyme (Wicklow, Ireland). All the chemicals were of analytical quality. For the preparation of the solution, ultrapure water (Milli-Q; Millipore, Billerica, MA, USA) was used. For bread production, white wheat flour type 500 was obtained from Žito d.o.o. Quality parameters of wheat flour: dry matter: 854.9 g/kg; water: 14.52%; ash: 0.535% on a dry basis; protein content: 13.6%; wet gluten: 31.4%; dry gluten: 10.6%; gluten index (GI): 89; enzyme activity (falling number): 366 s.

### 3.2. Preparation of Bioprocessed Spelt Flour

Germination of spelt seeds (*Triticum spelta* L. cv. Ostro) occurred under abiotic stress specified as darkness, at 25 °C for 144 h, with the addition of 25 mM NaCl after 48 h and 50 mM sorbitol after 96 h of germination according to Mencin et al. [11]. After germination, the spelt seeds with sprouts were lyophilised at −50 °C and 4.0 Pa (SP Scientific, AdVantage Pro, New York, NY, USA) and milled with a laboratory grinder (A11 basic, IKA^®^ Works, Staufen, Germany) (particle size < 0.25 mm). The lyophilized germinated spelt flour was then fermented for 72 h at 30 °C under static conditions in a Roto-Therm incubated rotator (Benchmark, Edison, NJ, USA), a sample-to-saline ratio of 1:1.5 (10 g:15 mL) and the addition of 0.75 mL of *Saccharomyces cerevisiae* inoculum (3% (*v*/*v*) inoculum) at an initial optical density of 1.6, corresponding to 2 × 10^8^ cfu/mL (hereinafter referred to as “germinated + fermented” flour) [9]. Enzymatic treatment of lyophilized germinated spelt flour was performed at 40 °C for 4 h, with the addition of cellulase (25 U/g DW), xylanase (5 U/g DW), feruloyl esterase (10 U/g DW), protease (50 U/g DW) and α-amylase (50 U/g DW) (hereinafter referred to as “germinated + enzymatic treated” flour) [12]. The bioprocessed spelt flours were lyophilised and stored at −20 °C. Since the quality and sensory characteristics of the bread with added “germinated + enzymatic treated” spelt flour was still unacceptable for average consumer (Figure S3), pasteurisation of that flour was performed at 85 °C for 30 min.

### 3.3. Bread-Making Procedure

The bread was made according to a white wheat bread recipe that served as a control bread and was made from 1000 g of wheat flour, 600 mL of water, 7.5 g of instant baker’s yeast, 19 g of salt and 25 mL of sunflower oil. The ingredients were mixed, and the dough was then fermented for 30 min. The dough was then divided and transferred to a proofing chamber, where the dough fermented again for 45 min at 30 °C and 85% relative humidity. Baking of the bread started with a dosage of 0.5 L of 180 °C hot steam, which was automatically dosed in the oven (Miwe Michael Wenz GmbH, Arnstein, Germany). The baking regime was as follows: 5 min at 230 °C, 20 min at 190 °C and 4 min at 200 °C. The bread was cooled for 3 h at room temperature. Depending on the type of bioprocessed bread to be produced, the commercial wheat flour was replaced by an appropriate proportion of bioprocessed spelt flour, taking into account a correction of the dry matter content. All other ingredients in the recipe and bread-making protocol were the same as for the control bread. Different bioprocessed breads were prepared as described in Table 5.

Bread quality characterisation and sensory evaluation were performed on all breads. With pasteurisation, we wanted to increase the percentage of added bioprocessed flour to the bread recipe while maintaining the quality of the bread. The selection for analysis of the phenolic content and antioxidant activity of breads included a control bread and four bioprocessed breads with the best quality and sensory properties (GEB1, GEB5P, GFB2.5, GFB5).

### 3.4. Rheological Properties of the Dough

The effect of adding 2.5% and 5% “germinated + fermented” spelt flour to wheat flour compared to the control flour (wheat flour) was investigated since these two breads had acceptable sensory quality. Farinographic and extensiographic properties were examined using a Farinograph and Extensiograph (Brabender, Duisberg, Germany) according to the AACC 54-21 and AACC 54-10.01 methods, respectively [43]. The farinographic parameters of the dough were the following: water absorption, dough development time, stability time, degree of softening (12 min after maximum) and farinograph quality number. The measured extensiographic parameters of the dough were energy (area under the curve), resistance to extension, maximum resistance to extension and extensibility.

### 3.5. Bread Quality Characterisation

Bread quality characterisation was performed after 3 h of bread cooling. The bread weights were taken, together with bread volumes measured using the rapeseed displacement method (AACC method 10-05.01). Specific volume was calculated as the ratio between the bread volume (mL) and weight (g).

The crust colour of the breads was measured with a Minolta Chroma Meter CR-400 (Konica Minolta, Japan). The instrumental colour parameters (five replicates on the same bread) of lightness (L*), redness (a*), and yellowness (b*) were determined. The total colour change (ΔE) between the control and breads enriched with bioprocessed spelt flour was calculated by the following formula: $\Delta E=\sqrt{{\Delta L}^{2}+{\Delta a}^{2}+{\Delta b}^{2}}$ [44].

Crumb firmness was determined by a texture analyser (TA.XT Plus; Stable Micro Systems Ltd., Surrey, UK) with a 36 mm diameter compression plate. For the texture profile analysis, the samples were compressed to 40% (10 mm) of their original thickness at a crosshead speed of 10 mm/s. Two slices taken from the centre of every bread sample, each 2.5 cm thick, were placed under an aluminium compression plate, and the force required for penetration was measured [45]. Three measurements were made for every slice.

### 3.6. Sensory Evaluation of Bread

The sensory evaluation was carried out on bread samples with the different percentages of bioprocessed wholegrain spelt flour 3 h after baking. The slice of each type of bread (2 cm thick) was numbered and served to each panellist under normal (daylight) illumination, and water was provided for rinsing between samples. The panel consisted of five trained assessors (aged between 31 and 55 years) who evaluated the bread’s overall acceptability (summarised assessment of all features, overall impression). In the sensory analysis of the bread, the external appearance of the bread was evaluated first (shape and appearance of the bread; appearance and colour of the crust). A slice of bread was then cut and the appearance of the crumb (porosity, colour), elasticity and stickiness of the crumb, crispness of the crust, odour and taste were evaluated. The individual parameters were scored as the intensity of the respective parameter compared to the reference sample (control bread). The score of all parameters for the reference sample was 0. Scores ranged from −3 to +3 points, and a positive score did not necessarily mean a positive description of a single parameter, just a more intensively expressed characteristic. If necessary, the panel supplemented the numerical score with a description of the specific properties.

### 3.7. Extractable and Bound Phenolics Extraction

The extraction of extractable and bound phenolic compounds was carried out as previously described by Mencin et al. [11]. Shortly, 1 g of the homogenised and lyophilised bread samples were mixed with 9 mL of absolute methanol. The mixture was shaken for 2 h in the dark at room temperature. Then the mixture was centrifuged at 9793.9× *g* for 10 min at 10 °C and filtered (pore size 0.45 µm). The methanol was evaporated off in a vacuum evaporator (HT-4 series II GeneVac Technologies, Ipswich, UK) at 40 °C, and the residues were resuspended in water. These filtered supernatants contained extractable phenolics. After methanol extraction, the remaining solid residues were then hydrolysed with 20 mL of 2 M sodium hydroxide and shaken in the dark for 4 h at room temperature. The hydrolysed samples were acidified to pH 3 with the addition of concentrated formic acid. These filtrated hydrolysates contained bound phenolics.

In addition, solid phase extraction (SPE) was used to purify the extracts and concentrate the phenolics and was performed according to Mencin et al. [11]. Firstly, the SPE cartridges (Strata-X RP; 100 mg; Phenomenex) were preconditioned with 3 mL of absolute methanol, followed by 3 mL of water. Supernatants containing extractable phenolics (5 mL) and hydrolysates containing bound phenolics (8 mL) were then added to the cartridges. The SPE cartridges were washed with 4 mL water and vacuum-dried for 2 min. Finally, we eluted the phenolics from the cartridges with 2 mL of 70% aqueous methanol. The obtained methanolic eluates represented the corresponding extractable and bound phenolic fractions. Purified samples were stored at 2 °C until analysis.

### 3.8. Total Phenolic Content

The TPCs in bread were evaluated using the Folin–Ciocalteu assay as described by Mencin et al. [11]. Briefly, the diluted bread extractable or bound fraction, water and Folin–Ciocalteu reagent were mixed. After incubation for 5 min, a sodium carbonate solution (20%, *w*/*v*) was added. After 1 h, the absorbance was measured in a 1 cm cuvette at 765 nm on a UV–visible spectrophotometer (model 8453; Hewlett Packard, Waldbronn, Germany). Final data were expressed as mg Trolox equivalents per g dry weight (mg TE/g DW).

### 3.9. Phenolic Profile

Reversed-phase HPLC-MS/MS analysis was used to quantify the individual phenolics (extractable and bound) in the bread samples. The HPLC system (Thermo Dionex system; Thermo Scientific, San Jose, CA, USA) equipped with an UV detector set at 280 nm and 310 nm was used. Chromatographic separation was carried out using a C18 column (Gemini C18; 150 mm × 4.6 mm; 3 µm; Phenomenex, Torrance, CA, USA). All of the individual phenolic compounds were identified using a mass spectrometer (LTQ XL linear ion trap mass spectrometer; Thermo Fisher Scientific, San Jose, CA, USA), with electrospray ionisation, operating in negative ionisation mode. Identification of phenolics was confirmed by comparisons of their UV-VIS spectra and MS spectra and retention times with external standards, followed by fragmentation, as fully described in the study of Mencin et al. [11]. Concentrations of *p*-coumaric, *trans*-ferulic, caffeic and *p*-hydroxybenzoic acids and gallocatechin were calculated according to peak areas, as compared to calibration curves with the corresponding standard. Concentrations were expressed as µg per g bread DW (µg/g DW). One peak was tentatively identified as the *cis*-isomer of ferulic acid because data from HPLC-MS analysis showed mass spectra that were nearly identical to those of *trans*-ferulic acid, so it was quantified using the calibration curve of *trans*-ferulic acid. For compounds identified without standards, quantification was performed using similar compounds as standards. Apigenin hexoside pentoside (I, II, III) and an unknown C-glycosyl derivative were quantified according to *p*-coumaric acid.

### 3.10. DPPH^•^ Radical Scavenging Activity

Antioxidant activity against the DPPH^•^ radical was determined as described in our previous publication [11]. Briefly, a 0.2 mM DPPH^•^ solution in 99.9% methanol was added to the appropriate diluted bread extracts. After 1 h, the decrease in absorbance was measured at 520 nm using a UV–visible spectrophotometer (model 8453; Hewlett Packard, Waldbronn, Germany). Methanol (99%) was used as a blank. The reference solution consisted of 0.2 mM DPPH^•^ solution and 99% methanol at a ratio of 1:1 (*v*:*v*). The DPPH^•^ radical scavenging activity (referred to as DPPH in Figures) was expressed as mg TE/g DW.

### 3.11. Statistical Analysis

All of the analyses were carried out in two parallel runs on two separate extractions. The results are presented as the mean ± standard deviation (SD). Statistical assessment was performed with the SPSS programme for Windows (version 22). The results were analysed using one-way ANOVA. The significance of the differences between the means was determined using Duncan’s post-hoc test with the significant difference at *p* < 0.05. Principal component analysis (PCA) was applied using OriginPro 2015 to analyse the contribution of all studied parameters to the overall quality of the different bread samples.

## 4. Conclusions

In conclusion, breads with up to 5% of “germinated + fermented” spelt flour showed potential for further research due to their improved nutritional value with still acceptable technological and sensory characteristics. Breads GFB30, GEB30 and GEB10P were unacceptable in terms of quality parameters due to the excessive activity of enzymes present in the bioprocessed flour. In GEB1 and GEB5P breads, there was a significant improvement in specific volume. A higher percentage of added bioprocessed spelt flour darkened the colour of the bread.

The highest content of extractable and bound TPCs was observed in GEB5P and GFB5 breads, and the greatest ability to scavenge DPPH^•^ radicals was also observed in GEB5P and GFB5 breads for extractable and bound fractions. The control bread had the lowest content of extractable and bound TPCs and, consequently, showed the lowest antioxidant activity. The predominant phenolic acid identified in the breads was *trans-*Ferulic acid, and more than 98% of total *trans-*ferulic acid was present in bound form. The GEB5P bread showed the highest increase in the content of extractable and bound *trans*-ferulic acid. We can assume that *trans*-ferulic and *p*-coumaric acids are the main contributors to the TPC and antioxidant activity of extractable and bound fractions.

The incorporation of bioprocessed spelt into wheat flour affected the rheological behaviour of the blends. Flour replacement (from 2.5 to 5%) by “germinated + fermented” spelt slightly lowered the water absorption capability and worsened the dough handling properties.

Evaluation of the bread-making process in terms of quality, sensory and especially nutritional properties confirmed that the addition of 2.5% or 5% “germinated + fermented” spelt flour is a promising and interesting ingredient for the formulation of bakery products, avoiding the use of enzymatic improvers and having a positive impact on consumer acceptance and facilitating the adoption of a “clean label”. It is crucial that consumers are informed about the presence of health-promoting substances in bioprocessed breads since this may improve the bread’s acceptability and, consequently, market potential. Our results indicate that the addition of bioprocessed flour significantly increases the phenolic content and antioxidant activity of bread. However, it is also important to determine whether these antioxidants are truly bioaccessible in the bioprocessed breads and, consequently, bioavailable for utilisation so that they can have beneficial health effects.

## Figures and Tables

**Table 1:** Farinographic and extensiographic parameters of control (wheat) dough and wheat dough enriched with 2.5% (GFB2.5) and 5% (GFB5) of »germinated + fermented« spelt flour.

		Dough		
		Control	GFB2.5	GFB5
Farinographic parameters	Water absorption (%)	60.1	56.8	56.0
	Development time (min)	2.0	1.7	1.5
	Stability time (min)	3.3	1.8	3.1
	Degree of softening (12 min after max.) (FU)	48	159	180
	Farinograph quality number	48	28	31
Extensiographic parameters *	Energy (cm^2^)	88	38	25
	Resistance to extension (BU)	404	163	116
	Maximum resistance to extension (BU)	517	179	119
	Extensibility (mm)	129	144	140

* Proving time: after 135 min.

**Table 2:** Quality parameters of bioprocessed breads compared to the control bread.

Bread	Specific Volume (mL/g)	Lightness (L*)	Redness (a*)	Yellowness (b*)	ΔE	Firmness (g Force)
Control	2.57 ± 0.11 ^c^	69.82 ± 1.31 ^f^	6.69 ± 0.73 ^a^	33.74 ± 0.70 ^cd^		352.24 ± 39.91 ^a^
GFB30	1.37 ± 0.19 ^a^	28.46 ± 0.96 ^a^	10.16 ± 0.56 ^b^	12.29 ± 0.88 ^a^	46.72	/
GEB30	/	/	/	/	/	/
GFB5	2.00 ± 0.16 ^b^	52.09 ± 0.79 ^c^	14.18 ± 0.58 ^f^	33.71 ± 0.24 ^cd^	19.25	744.89 ± 70.60 ^b^
GEB5	1.98 ± 0.09 ^b^	52.75 ± 1.17 ^c^	11.51 ± 1.24 ^cd^	33.05 ± 0.27 ^c^	17.75	/
GFB2.5	2.28 ± 0.09 ^bc^	57.37 ± 0.42 ^d^	12.66 ± 0.24 ^de^	36.09 ± 0.26 ^e^	14.00	420.83 ± 27.82 ^a^
GEB1	2.99 ± 0.07 ^d^	60.82 ± 2.63 ^e^	11.00 ± 0.64 ^bc^	37.21 ± 0.41 ^f^	10.56	/
GEB10P	2.23 ± 0.30 ^bc^	47.43 ± 1.29 ^b^	14.32 ± 0.59 ^f^	31.07 ± 0.62 ^b^	23.81	/
GEB5P	3.03 ± 0.18 ^d^	53.50 ± 1.70 ^c^	13.64 ± 0.60 ^ef^	34.27 ± 0.46 ^d^	17.75	/

Data are means ± SD. Means with different letters within a column indicate statistically significant differences (*p* < 0.05; Duncan’s Multiple Range Test). Control: white wheat bread; GFB30: wheat bread supplemented with 30% “germinated + fermented” spelt flour; GEB30: wheat bread supplemented with 30% “germinated + enzymatic treated” spelt flour; GFB5: wheat bread supplemented with 5% “germinated + fermented” spelt flour; GEB5: wheat bread supplemented with 5% “germinated + enzymatic treated” spelt flour; GFB2.5: wheat bread supplemented with 2.5% “germinated + fermented” spelt flour; GEB1: wheat bread supplemented with 1% “germinated + enzymatic treated” spelt flour; GEB10P: wheat bread supplemented with 10% pasteurised (5 min, 85 °C) “germinated + enzymatic treated” spelt flour; GEB5P: wheat bread supplemented with 5% pasteurised (30 min, 85 °C) “germinated + enzymatic treated” spelt flour. /: could not be detected.

**Table 3:** Sensory attributes of bioprocessed breads, with the control bread serving as a basis for comparison, and the sensory attributes rated with scores from −3 to +3.

Attributes	Control	GFB5	GFB2.5	GEB1	GEB5P
Overall quality	0	−1.5 (smaller bread volume)	−2	−1 (irregular shape, cracked)	−1 (irregular shape, cracked)
Crust appearance	0 (crust cracks)	−1 (crust does not crack, appropriate thick)	−1	−0.5 (less cracked bread)	−1 (does not crack)
Crust colour	0	2	1.5	1	1.5
Crust crispiness	0	0.5	0.5	2	2 (crispy, but the crumb is not suitable for eating)
Crumb porosity	0	−0.5 (uniform pores)	−0.5 (smaller air bubbles, more uniform porosity, less distinct water ring)	2 (larger air bubble, pores, uneven porosity)	2 (larger air bubble, pores, uneven porosity)
Crumb colour	0	1	0.5	0.5	2 (darker crumb)
Elasticity	0	−0.5 (it returns to its original shape quite fast)	−1 (returns to its original shape more slowly)	−3 (does not return to its original shape)	−2.5 (does not return to its original shape)
Stickiness	0	0.5 (less sticky than GFB2.5)	1.5 (a bit sticky)	3 (extremely sticky)	3 (unacceptable)
Odour	0	0.5 (slightly more distinct smell)	0.5 (slightly more intense smell after spelt)	−0.5 (indistinct smell)	−0.5 (indistinct smell)
Taste	0	1 (slightly more distinct, after spelt, less sour, after cheese-yeast extract)	1 (slightly more distinct, after spelt, crust- taste after cheese and caramel)	/(not suitable for consuming)	−2 (sweet taste, uncharacteristic for bread)

Control: white wheat bread; GFB5: wheat bread supplemented with 5% “germinated + fermented” spelt flour; GFB2.5: wheat bread supplemented with 2.5% “germinated + fermented” spelt flour; GEB1: wheat bread supplemented with 1% “germinated + enzymatic treated” spelt flour; GEB5P: wheat bread supplemented with 5% pasteurised (30 min, 85 °C) “germinated + enzymatic treated” spelt flour.

**Table 4:** Contents of individual phenolic compounds in the extractable and bound fractions of control and bioprocessed breads.

Phenolics Content (µg/g DW)		Breads				
		Control	GEB1	GEB5P	GFB2.5	GFB5
***trans*-Ferulic acid**	**Extractable**	0.56 ± 0.04 ^a^	0.75 ± 0.03 ^b^	2.35 ± 0.08 ^e^	1.49 ± 0.08 ^c^	2.10 ± 0.18 ^d^
	**Bound**	45.88 ± 3.12 ^A^	69.31 ± 3.68 ^B^	108.60 ± 5.06 ^E^	81.73 ± 3.41 ^C^	99.31 ± 4.46 ^D^
***cis*-Ferulic acid**	**Extractable**	0.16 ± 0.02 ^a^	0.22 ± 0.02 ^b^	0.56 ± 0.01 ^e^	0.33 ± 0.02 ^c^	0.39 ± 0.02 ^d^
	**Bound**	18.78 ± 0.18 ^B^	24.47 ± 1.54 ^C^	27.15 ± 0.56 ^D^	15.98 ± 0.28 ^A^	27.03 ± 1.06 ^D^
***p*-Coumaric acid**	**Extractable**	0.12 ± 0.00 ^a^	0.11 ± 0.01 ^a^	0.15 ± 0.01 ^c^	0.13 ± 0.00 ^b^	0.17 ± 0.01 ^d^
	**Bound**	1.25 ± 0.12 ^A^	2.07 ± 0.11 ^B^	6.86 ± 0.49 ^E^	2.71 ± 0.07 ^C^	5.44 ± 0.40 ^D^
**Caffeic acid**	**Extractable**	0.06 ± 0.01 ^a^	0.09 ± 0.01 ^b^	0.10 ± 0.01 ^b^	0.07 ± 0.00 ^a^	0.12 ± 0.01 ^c^
	**Bound**	0.30 ± 0.02 ^A^	0.46 ± 0.04 ^B^	1.89 ± 0.14 ^C^	0.51 ± 0.07 ^B^	1.80 ± 0.06 ^C^
***p*-Hydroxybenzoic acid**	**Extractable**	0.27 ± 0.01 ^a^	0.30 ± 0.02 ^a^	0.37 ± 0.03 ^b^	0.44 ± 0.02 ^c^	0.70 ± 0.02 ^d^
	**Bound**	3.76 ± 0.13 ^A^	4.67 ± 0.14 ^B^	8.23 ± 0.18 ^E^	5.25 ± 0.26 ^C^	6.04 ± 0.13 ^D^
**Apigenin hexoside pentoside I**	**Extractable**	0.28 ± 0.02 ^a^	0.32 ± 0.01 ^b^	0.53 ± 0.02 ^d^	0.33 ± 0.00 ^b^	0.38 ± 0.02 ^c^
	**Bound**	0.89 ± 0.02 ^A^	1.29 ± 0.01 ^C^	2.26 ± 0.08 ^D^	1.00 ± 0.01 ^B^	2.36 ± 0.06 ^D^
**Apigenin hexoside pentoside II**	**Extractable**	0.08 ± 0.01 ^a^	0.10 ± 0.01 ^b^	0.18 ± 0.01 ^d^	0.11 ± 0.00 ^b^	0.14 ± 0.01 ^c^
	**Bound**	0.22 ± 0.01 ^A^	0.49 ± 0.01 ^C^	1.12 ± 0.05 ^D^	0.39 ± 0.00 ^B^	1.28 ± 0.03 ^E^
**Apigenin hexoside pentoside III**	**Extractable**	0.30 ± 0.02 ^b^	0.33 ± 0.01 ^c^	0.41 ± 0.02 ^d^	0.28 ± 0.01 ^b^	0.25 ± 0.01 ^a^
	**Bound**	1.41 ± 0.04 ^A^	1.67 ± 0.03 ^B^	2.38 ± 0.10 ^C^	1.41 ± 0.01 ^A^	2.43 ± 0.08 ^C^
**Unknown C-glycosyl derivative**	**Extractable**	0.10 ± 0.00 ^b^	0.09 ± 0.00 ^b^	0.10 ± 0.01 ^b^	0.07 ± 0.01 ^a^	0.07 ± 0.01 ^a^
	**Bound**	0.39 ± 0.01 ^A^	1.01 ± 0.01 ^B^	2.69 ± 0.18 ^C^	1.09 ± 0.05 ^B^	3.17 ± 0.03 ^D^

Data are means ± SD (*n* = 6). Different small letters within a row (phenolic) indicate significant differences between the extractable phenolic contents (*p* < 0.05; Duncan’s Multiple Range Test). Different capital letters within a row indicated significant differences between the bound phenolic contents (*p* < 0.05; Duncan’s Multiple Range Test). Control: white wheat bread; GEB1: wheat bread supplemented with 1% “germinated + enzymatic treated” spelt flour; GEB5P: wheat bread supplemented with 5% pasteurised (30 min, 85 °C) “germinated + enzymatic treated” spelt flour; GFB2.5: wheat bread supplemented with 2.5% “germinated + fermented” spelt flour; GFB5: wheat bread supplemented with 5% “germinated + fermented” spelt flour.

**Table 5:** Dough formulation of white wheat bread (control) and wheat bread enriched with bioprocessed spelt flour.

Ingredients	Control	GFB30	GEB30	GFB5	GEB5	GFB2.5	GEB1	GEB10P	GEB5P
white wheat flour (g)	1000	700	700	950	950	975	990	900	950
bioprocessed flour (g)									
germinated + fermented	/	300	/	50	/	25	/	/	/
germinated + enzymatic treated	/	/	300	/	50	/	10	100	50
water (mL)	600	600	600	600	600	600	600	600	600
instant baker’s yeast (g)	7.5	7.5	7.5	7.5	7.5	7.5	7.5	7.5	7.5
salt (g)	19	19	19	19	19	19	19	19	19
sunflower oil (mL)	25	25	25	25	25	25	25	25	25

Control: 100% white wheat flour; GFB30: white wheat flour plus 30% “germinated + fermented” spelt flour bread; GEB30: white wheat flour plus 30% “germinated + enzymatic treated” spelt flour bread; GFB5: white wheat flour plus 5% “germinated + fermented” spelt flour bread; GEB5: white wheat flour plus 5% “germinated + enzymatic treated” spelt flour bread; GFB2.5: white wheat flour plus 2.5% “germinated + fermented” spelt flour bread; GEB1: white wheat flour plus 1% “germinated + enzymatic treated” spelt flour bread; GEB10P: white wheat flour plus 10% pasteurised (30 min, 85 °C) “germinated + enzymatic treated” spelt flour bread; and GEB5P: white wheat flour plus 5% pasteurised (30 min, 85 °C) “germinated + enzymatic treated” spelt flour bread.

## Data Availability

All data are contained in this article and Supplementary Materials.

## Supplementary Materials

The following supporting information can be downloaded at: https://www.mdpi.com/article/10.3390/molecules28083428/s1, Figure S1: Farinograms of control (wheat) dough and wheat dough enriched with 2.5% (GFB2.5) and 5% (GFB5) “germinated + fermented” spelt flour; Figure S2: Extensiograms of control (wheat) dough and wheat dough enriched with 2.5% (GFB2.5) and 5% (GFB5) “germinated + fermented” spelt flour; Figure S3: Photographs of breads prepared from white wheat flour (control), with 5% of »germinated + enzymatic treated« spelt flour (GEB5), with 10% of pasteurised »germinated + enzymatic treated« spelt flour (GEB10P), with 30% of “germinated + enzymatic treated” (GEB30) and with 30% of “germinated + fermented” spelt flour (GFB30).
