# Supplementary material for: Bioprocessed Wholegrain Spelt Flour Improves the Quality and Physicochemical Characteristics of Wheat Bread
Source: Molecules. 2023 Apr 13;28(8):3428. doi: 10.3390/molecules28083428 (PMC10146097; doi:10.3390/molecules28083428)
Supplement: Supplementary file 1 [file molecules-28-03428-s001.zip › molecules-2342922-supplementary.pdf]

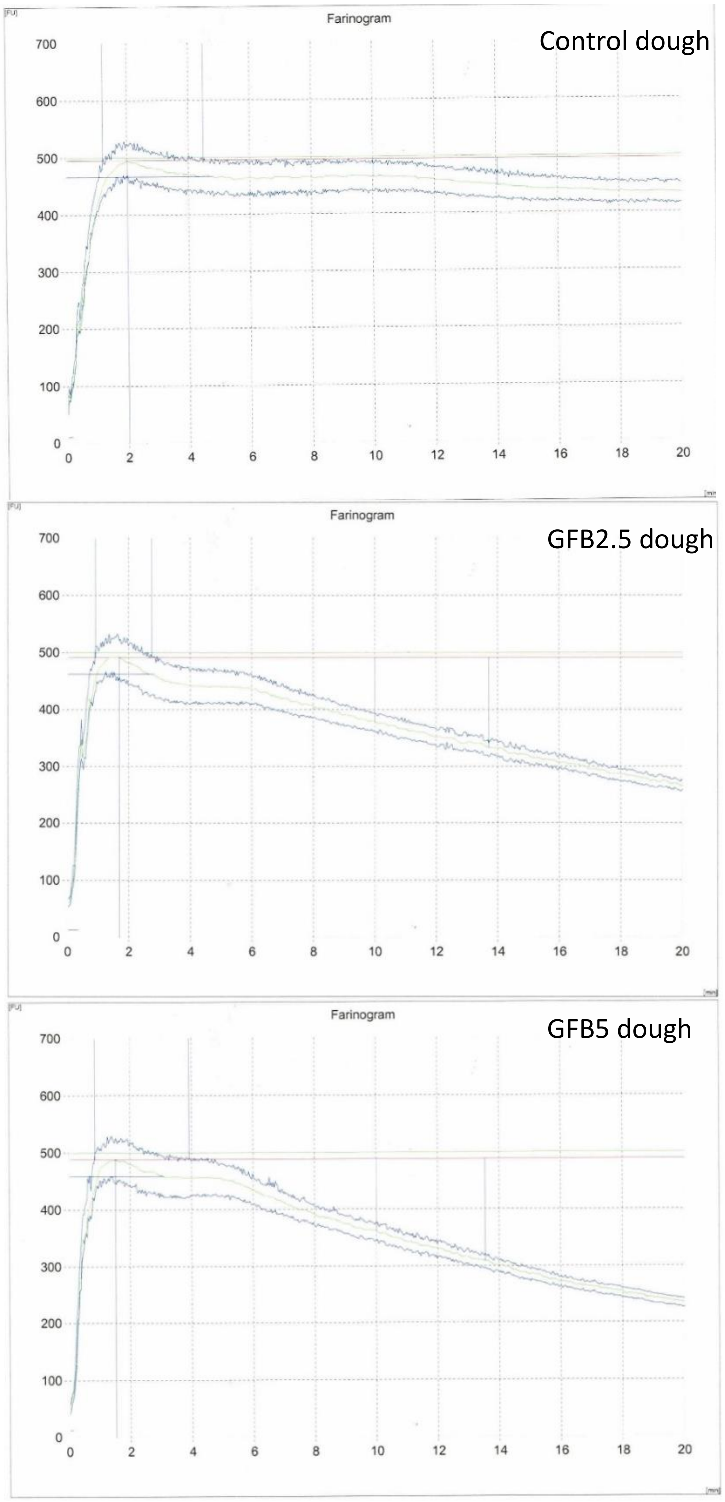

Figure S1. Farinograms of control (wheat) dough and wheat dough enriched with 2.5% (GFB2.5) and 5% (GFB5) "germinated + fermented" spelt flour.

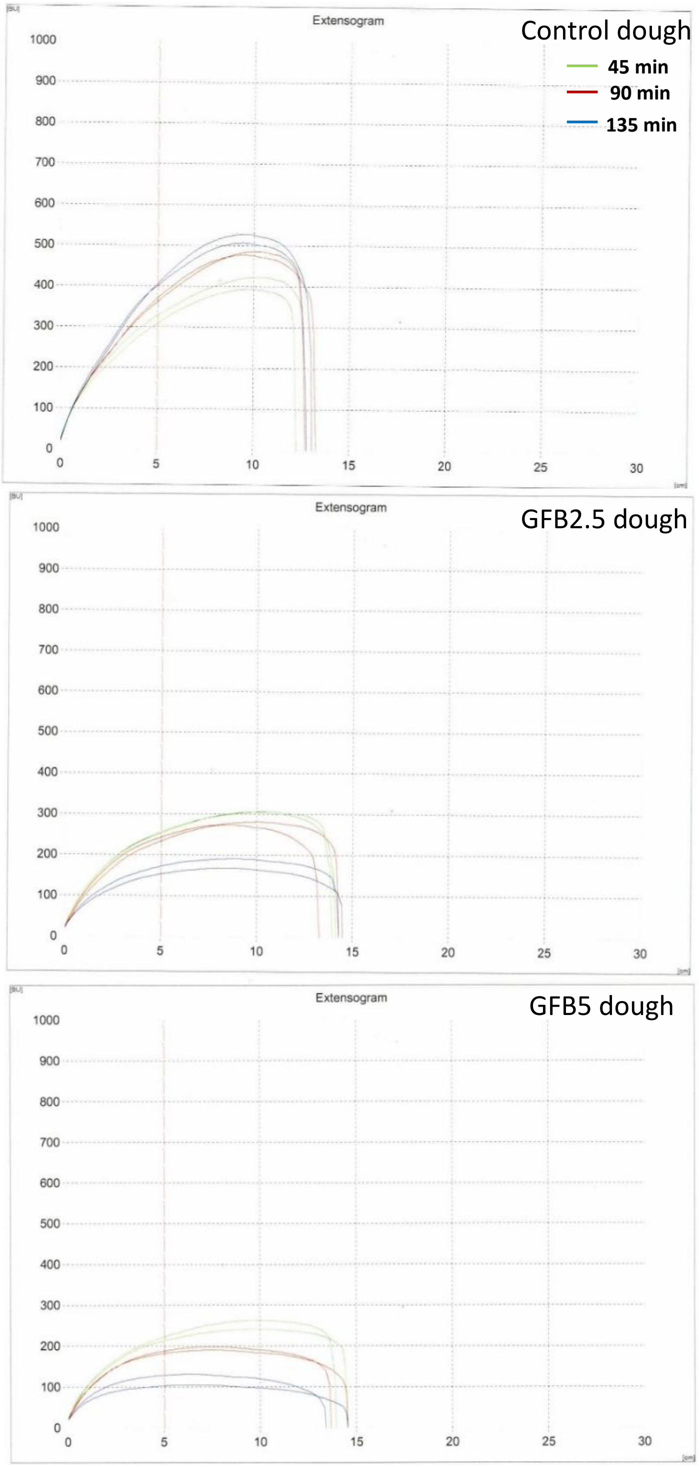

Figure S2. Extensiograms of control (wheat) dough and wheat dough enriched with 2.5% (GFB2.5) and 5% (GFB5) “germinated + fermented” spelt flour.

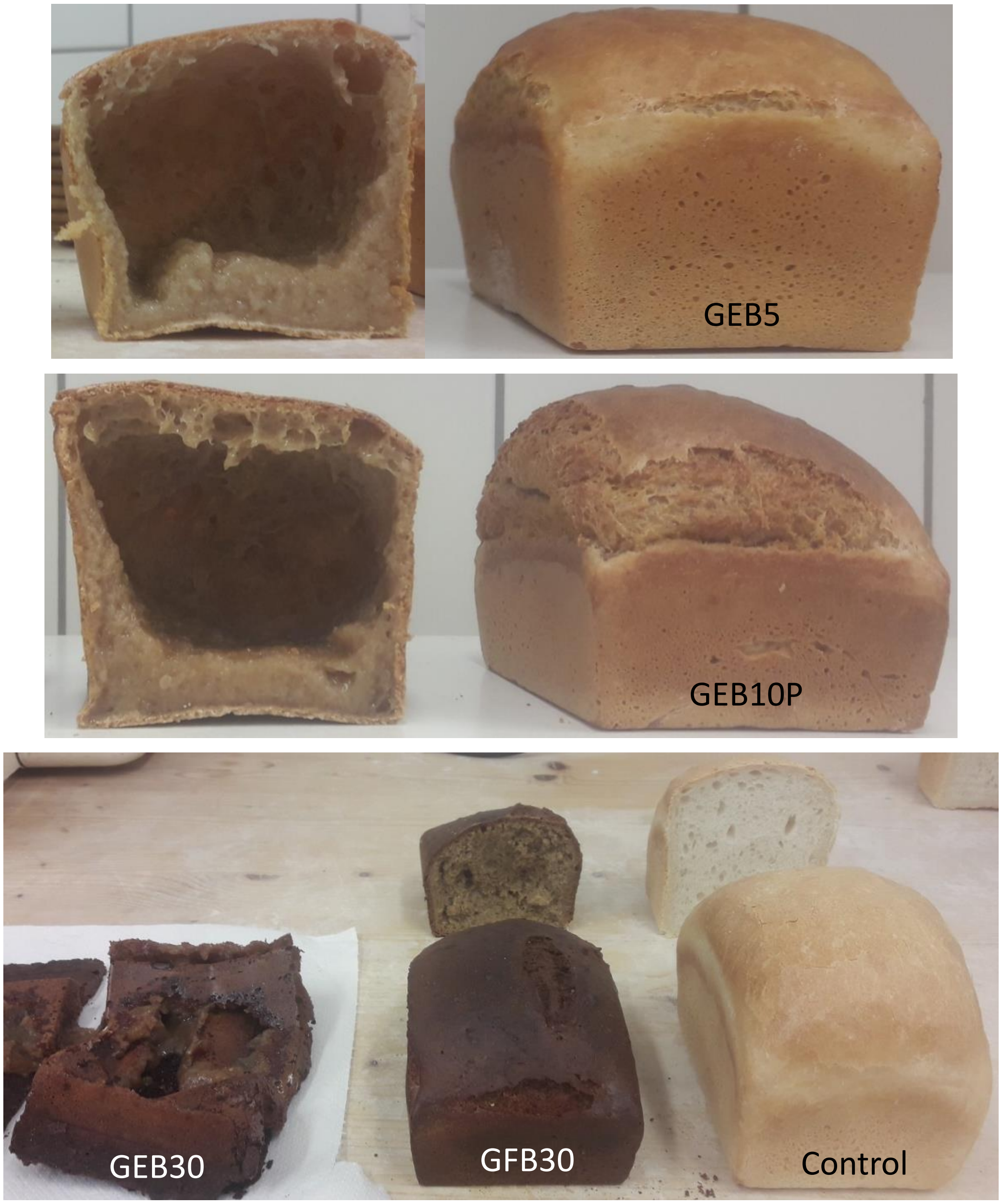

Figure S3. Photographs of breads prepared from white wheat flour (control), with 5% of »germinated + enzymatic treated« spelt flour (GEB5), with 10% of pasteurized »germinated + enzymatic treated« spelt flour (GEB10P), with 30% of "germinated + enzymatic treated" (GEB30) and with 30% of "germinated + fermented" spelt flour (GFB30).
